# Small-pox in Texas

**Published:** 1891-01

**Authors:** 


### EDITORIAL DEPARTMENT.

F. E. DANIEL, M. D., EDITOR AND PUBLISHER.

THIS JOURNAL, by unanimous vote of the members, was made the Official Organ of the Austin District Medical Society.

COLLABORATORS:

*Dr. R. M. Swearingen, Austin.*
*Prof. B. E. Hadra, M. D., Galveston.*
*Prof. Geo. Cupples, M. D., San Antonio.*
*Dr. T. C. Osborn, Cleburne, Texas.*
*Dr E. J. Doering, Chicago.*
*Dr. E. J. Beall, Fort Worth.*
*Dr Odo Betz, Germany.*
*Prof. W. B. Rogers, M. D., Memphis.*
*Dr. J. W. McLaughlin, Austin.*
*Dr. T. J. Tyner, Austin.*
*Prof. J. F. Y. Paine, M. D., Galveston.*
*Dr. R. H. L. Bibb, Mexico.*
*Dr. T. J. Bennett. Austin.*
*Dr. Bat Smith, Wharton.*
*Dr. E. Meierhof, New York.*
*L. H. Luce, M. D., West Tisbury, Mass.*

### SMALL-POX IN TEXAS.

### The Necessity for a State Board of Health Demonstrated.

The Governor has isued the following proclamation:

*To whom these presents shall come:*

WHEREAS, It appears from reports made to me that the contagious disease of small-pox has made its appearance at various points within the State, at which the local authorities state they are unable to cope with or control said disease; and

WHEREAS, There appears to be a conflict in the general quaraotine laws whereby great confusion and hindrance to the commerce and trade of the State may be incurred, unless the State of Texas, acting by its health officer, shall, by proper rules and regulations, take control of all local quarantines;

THEREFORE, By virtue of the powers conferred upon me, and the requirements thereunder of the quarantine laws made and provided, commencing at Art. 5090 of our Revised Statutes and subsequent enactments, I declare a quarantine against all places wherein the said contagious disease of small-pox may now, or during the pendency of this proclamation, exist, and direct that the state health officer shall inquire into the condition of affairs at all such places, and shall make such rules and regulations to enforce quarantine and control and stamp out the said disease, as under the law and the circumstances in each case may seem to him to be for the interest of the public.

In testimony whereof I hereunto subscribe my name
[L. S.] and cause the Seal of State to be affixed at Austin, this 2nd day of January, 1891.

[Signed] L. S. Ross, Governor.

By the Governor:
J. M. Moore, Secretary of State.

Can anything more forcibly demonstrate the necessity for a change in the present so-called "health system" of Texas? We have no health systsm; there is a qurantine law, and strict measures are enforced as to maritime quarantine,—and so far as it goes the law is good enough. But Texas needs something besides maritime quarantine; there is no law regulating internal quarantine; and there is little or no protection against invasion from Mexico. In all probability the infection now spreading in the interior of Texas was introduced from Mexico; we know that complaint has been made on the frontier that there was either no protection, or that it was not efficient, and that the disease was imported into Laredo via Mexico. Because of the want of a law to regulate internal quarantine, the people in one or two instances have taken the matter in hand and instituted, practically, "shot-gun" quarantine, notably at Marlin against Waco, some time since, and it has resulted in damage to trade and ill-feeling between neighboring towns, as well as to interruption of travel. In the instance referred to it brought about a conflict between the local authorities and the State government, and the governor had to go in person to enforce the raising of the blockade. Now, it seems, this thing is to be repeated at some dozen or more points in the State. The disease has broken out in Greer county, away up in the Panhandle, and in the interior, and in the Southwest, and at or near Houston and Galveston. In consequence of the absence of a law to regulate matters. the local authorities must act as they think best, and as that action is not always characterized by the highest evidence of intelligence, a conflict is bound to ensue. We heard of one so-called health officer who had been sent to deal with an outbreak of small-pox, and when asked why he did not cause a general vaccination, replied: "They have already been exposed and vaccination will only make it worse." He should be "bored for the simples;" but it is only mentioned in support of the above assertion.

It is to be hoped that the State Health Officer may be able to harmonize things and to stamp out the disease promptly.

The Legislature should promptly amend the law so as to obviate similar conflicts in the future, and no doubt his Excellency, Governor Hogg, will so recommend.

But, while they are about it, why not give the State a complete Board of Health? We have, as stated, a good quarantine service, as against foreign invasion. We would advocate letting that alone, as far as possible, and adding to it. Let them create a State Board of Health, and let the quarantine office be a member with a separate and especial function; but let him act jointly with other members in all other matters that pertain to a Board of Health. We regard the preservation of vital statistics of prime importance, and the Board should attend to it; interior sanitation should be a function, and the supervision of the sale of foods, liquors, drugs and medicines, etc., should be provided for by them. It has been pointed out repeatedly that a Board of Health, such as exists in twenty-six States, can be operated at less cost than has attended the administration of outside quarantine alone, in Texas in recent years. Therefore, from every consideration of right, duty, necessity and economy, we urge the creation of a Board of Health for Texas at an early day by the Legislature.

Our Committee on Medical Legislation, composed of some of the ablest medical men in Texas, have prepared a bill for the purpose of creating a Board of Health, and will present it in person soon after the Legislature meets. The bill provides that the Board of Health shall also be a Board of Medical Examiners to examine aplicants for license to practice, and the JOURNAL is informed that the bill will be presented to the Legislature, backed by petitions from every class of people from every section of Texas, to the number of twenty-five thousand or more. Will the Legislature heed this popular demand?

